# High-Performance Na-Ion Storage of S-Doped Porous Carbon Derived from Conjugated Microporous Polymers

**DOI:** 10.1007/s40820-019-0291-z

**Published:** 2019-07-17

**Authors:** Yuquan Li, Bin Ni, Xiaodan Li, Xianghui Wang, Dafeng Zhang, Qingfei Zhao, Jinliang Li, Ting Lu, Wenjie Mai, Likun Pan

**Affiliations:** 1 Shanghai Key Laboratory of Magnetic Resonance, School of Physics and Electronic Science, East China Normal University, 3663 N. Zhongshan Rd., Shanghai, 200062 People’s Republic of China; 2 Siyuan Laboratory, Guangdong Provincial Engineering Technology Research Center of Vacuum Coating Technologies and New Energy Materials, Department of Physics, Jinan University, Guangzhou, 510632 Guangdong People’s Republic of China; 3 School of Materials Science and Engineering, Liaocheng University, Liaocheng, 252000 Shandong People’s Republic of China; 4 Department of Chemical Engineering, School of Environmental and Chemical Engineering, Shanghai University, 99 Shangda Road, Shanghai, 200444 People’s Republic of China; 5 Testing and Analysis Centre, College of Chemistry and Materials Science, Shanghai Normal University, 100 Guilin Road, Shanghai, 200234 People’s Republic of China

**Keywords:** Conjugated microporous polymer, S-doped porous carbons, Na-ion batteries, Reaction mechanism

## Abstract

Na-ion batteries (NIBs) have attracted considerable attention in recent years owing to the high abundance and low cost of Na. It is well known that S doping can improve the electrochemical performance of carbon materials for NIBs. However, the current methods for S doping in carbons normally involve toxic precursors or rigorous conditions. In this work, we report a creative and facile strategy for preparing S-doped porous carbons (SCs) via the pyrolysis of conjugated microporous polymers (CMPs). Briefly, thiophene-based CMPs served as the precursors and doping sources simultaneously. Simple direct carbonization of CMPs produced S-doped carbon materials with highly porous structures. When used as an anode for NIBs, the SCs exhibited a high reversible capacity of 440 mAh g^−1^ at 50 mA g^−1^ after 100 cycles, superior rate capability, and excellent cycling stability (297 mAh g^−1^ after 1000 cycles at 500 mA g^−1^), outperforming most S-doped carbon materials reported thus far. The excellent performance of the SCs is attributed to the expanded lattice distance after S doping. Furthermore, we employed ex situ X-ray photoelectron spectroscopy to investigate the electrochemical reaction mechanism of the SCs during sodiation–desodiation, which can highlight the role of doped S for Na-ion storage.

**Electronic supplementary material:**

The online version of this article (10.1007/s40820-019-0291-z) contains supplementary material, which is available to authorized users.

## Highlights

- S-doped porous carbons (SCs) derived from conjugated microporous polymers were synthesized for Na-ion batteries.
- The SCs exhibited a high capacity of 440 mAh g^−1^ at 50 mA g^−1^ and excellent cycling performance.
- Ex situ X-ray photoelectron spectroscopy was used to investigate the electrochemical reaction mechanism of the SCs.

**Electronic supplementary material:**

The online version of this article (10.1007/s40820-019-0291-z) contains supplementary material, which is available to authorized users.

## Graphical abstract

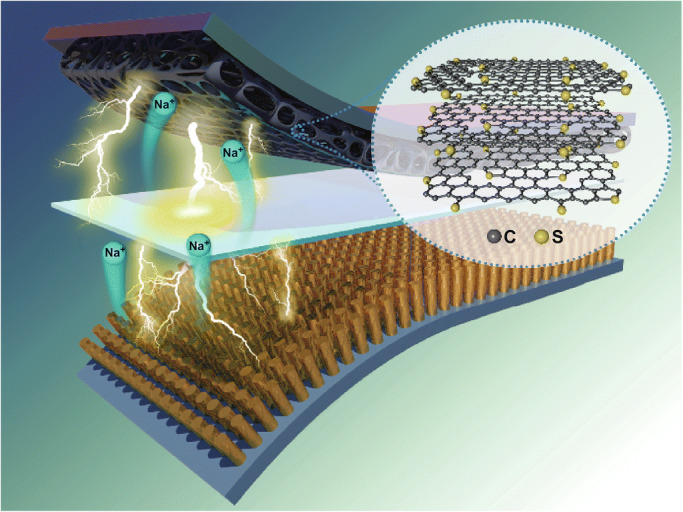

## Introduction

Recently, Li-ion batteries (LIBs) have seen tremendous progress owing to the significant development of electrical devices and electric vehicles [1–4]. However, the emerging demand for LIBs has resulted in a latent shortage of Li. Therefore, Na-ion batteries (NIBs), as one of the potential alternatives to LIBs, have attracted increasing research attention owing to the low cost and eco-friendliness of Na [5–8]. However, the radius of the Na ion is larger than that of the Li ion; therefore, the diffusion of Na ions is hindered in the NIB system. Hence, it remains a challenge to find a suitable accommodator to realize reversible and fast insertion–extraction of Na ions [9–11].

Carbonaceous materials are candidates for the anode of NIBs, owing to their abundance, superior conductivity, and outstanding stability [12, 13]. In numerous studies, carbonaceous materials have exhibited excellent cycling stability as the anode for NIBs [14–16]. However, owing to the low diffusion coefficient of Na, carbonaceous materials deliver a poor reversible capability [17, 18]. It is well known that introducing heteroatoms, such as S, can modify the structure and enhance the electrical conductivity of carbon materials [17, 19–21]. Additionally, S doping has been shown to improve the Na-ion storage capability of carbon materials by producing defects and pores [22] and increasing the interlayer distance [10, 23]. For example, by conducting pyrolysis of 1,4,5,8-naphthalenetetracarboxylic dianhydride in S steam, Li et al. [9] obtained S-doped disordered carbon with a high initial reversible capacity of 516 mAh g^−1^ at 20 mA g^−1^ for NIBs. Yang et al. [24] prepared S-doped N-rich carbon via thermolysis of N-rich carbon in an H_2_S/Ar atmosphere, which delivered excellent reversibility for Na-ion storage (350 mAh g^−1^ at 50 mA g^−1^). Hong et al. [25] obtained S-doped hard carbon through the molten salts method using S and Na_2_S_2_O_3_ powders as S sources, which exhibited a capacity of 200 mAh g^−1^ at 1000 mA g^−1^. These studies indicate that S exhibits high activity with a reversible electrochemical reaction for Na-ion storage when it is doped in carbonaceous materials. However, the methods used in previous studies for S doping suffer from several shortcomings, including toxic precursors, complex processes, and strict conditions. Additionally, the Na-ion storage capacity of the reported S-doped carbon must be improved for practical applications. Therefore, developing a facile approach for the preparation of high-performance S-doped carbon materials remains highly challenging [26].

As organic porous polymers, conjugated microporous polymers (CMPs), which are composed of lightweight elements and connected by inherent *π* conjugation and strong covalent linkage, are attracting attention [27, 28]. CMPs have a wide range of potential applications, including gas adsorption, catalysis [29], sensors [30], and energy storage [31–34], owing to their tunable and flexible structures [35, 36]. In particular, CMPs with a suitable porosity and heteroatom doping level can be obtained by tuning their porous architectures and functional groups, respectively, which are the ideal precursors for in situ heteroatom-doped carbons [37–39]. For example, Zhuang et al. [40] obtained B, N-co-doped porous carbons derived from CMPs and found that the rich B, N-doped porous carbons exhibited high catalytic performance for the oxygen reduction reaction (ORR). Hao et al. [41] prepared N-doped carbon materials derived from CMPs with a specific capacitance of 151 F g^−1^ at 0.1 A g^−1^ for a supercapacitor. Recently, Su et al. [42] synthesized N, S-co-doped carbon nanosheets through the activation of S-doped CMPs at different temperatures in an ammonia atmosphere, which exhibited high catalytic performance for the ORR. These aforementioned works offer a facile strategy for the in situ preparation of S-doped porous carbons (SCs) from CMPs and indicate the possibility of applying the SCs to the anodes of NIBs owing to their superior structure and electrochemical behaviors. However, relevant exploration has not been performed.

In this work, a S-containing thiophene-based CMP (SCMP) was prepared through a conventional solution phase reaction method and used as the precursor as well as the doping source for in situ preparation of SCs. Owing to the abundance and regular distribution of S atoms in the SCMP precursor, the S element was successfully doped into the carbon skeletons. The as-prepared SCs exhibited excellent Na-ion storage performance, indicating their potential for high-performance NIBs.

## Experimental

### Synthesis

In a typical procedure, 6.0 g of 1,2,4,5-benzenetetracarboxylic anhydride (PDA) and 20.0 g of phosphorus pentasulfide were dissolved in a xylene solution (150 mL). Then, the mixture was vigorously stirred at 120 °C for 7 days under a reflow process. After the reaction, the black powder was collected via filtration, and then the powder was further extracted using tetrahydrofuran in a Soxhlet apparatus for 3 days and dried at 80 °C for 12 h. The obtained SCMP was carbonized at different temperatures for 4 h with a heating rate of 5 °C min^−1^ in a N_2_ atmosphere. Subsequently, the samples were immersed in water at 70 °C overnight, followed by washing. The obtained products were dried at 60 °C for 6 h. To evaluate the economy of our SCs, the yield of polymerization and the quality after pyrolysis were examined. More than 3.7 g of SCMP was obtained after the polymerization of 6.0 g of PDA and purification through Soxhlet extraction. Finally, approximately 1.2 g of the sample was obtained after carbonization. PDA is a low-cost CMP precursor (~ $60 per kg); most organic units for preparing microporous polymers are more expensive. Additionally, most S-doped carbon materials used for SIBs employ powdered S and H_2_S as doping sources. During the pyrolysis of powdered S and H_2_S, a significantly larger amount of S is wasted in the tail gas compared with our pyrolysis process, which is environmentally unfriendly for large-scale production. Our approach is an economical and scalable method to obtain S-doped carbon for SIB applications.

### Materials Characterization

To study the bond structure of SCMP, the as-prepared polymer was characterized via Fourier transform infrared (FTIR) spectroscopy and ^13^C nuclear magnetic resonance (NMR) spectroscopy (Bruker AVANCE III 300 Spectrometer). Powder X-ray diffraction (XRD, Bruker) patterns were obtained using Cu Kα radiation (*λ* = 1.5406 Å). The morphologies and structures of the samples were observed via scanning electron microscopy (SEM, S-4800, Hitachi) and transmission electron microscopy (TEM, JEM-2010, JEOL). A multifunctional X-ray photoelectron spectrometer (XPS, ESCALAB 250XI, Thermo Scientific) was employed to analyze the chemical states and compositions of the samples. Raman spectra were obtained using a Raman spectrometer (RM-1000, Renishaw) with a laser having a wavelength of 632.8 nm. The carbon conversion rate of the SCMP was characterized via thermogravimetric analysis (TGA, TGA/DSC 1/1600HT, Metter), under heating from room temperature to 1000 °C with a heating rate of 10 °C min^−1^ in a N_2_ atmosphere. The N_2_ adsorption–desorption isotherms were obtained at 77 K using an Autosorb iQ2 system (Quantachrome Instruments), and the specific surface area and pore-size distribution were calculated from the adsorption data using the Brunauer–Emmett–Teller (BET) model.

### Electrochemical Testing

In a typical procedure, active materials, carbon black and carboxymethyl cellulose were mixed in water with a weight ratio of 8:1:1 for homogenous sizing. Then, the solution was coated onto Cu foil and dried at 120 °C for 24 h. The mass of the active materials for each electrode was approximately 1 mg cm^−2^. Electrochemical tests were conducted at atmospheric temperature in coin cells (C2032-type), which were assembled in an MBraun glovebox filled with Ar. In the coin cells, the counter electrode was Na metal foil, the separator was a glass fiber filter (Whatman), and the electrolyte was ethylene carbonate and propylene carbonate (1:1, w/w) with 1 M NaClO_4_. The cycling performance and galvanostatic charge/discharge curves of the electrodes were evaluated using a LAND 2001A battery test system in the voltage range of 0.005–3.0 V. Unless otherwise noted, cyclic voltammetry (CV) was performed using an Autolab electrochemical workstation (PGSTAT 204) at a sweep rate of 0.2 mV s^−1^. Electrochemical impedance spectroscopy (EIS) was performed using the same electrochemical workstation after 100 cycles in the frequency range of 0.1 Hz to 100 kHz.

## Results and Discussion

### Structural Characterizations

The typical fabrication method for the SCMP is illustrated in Fig. S1. The SCMP was polymerized by a PDA monomer with phosphorus pentasulfide in xylene for 7 days. During the process, phosphorus pentasulfide, which is a commonly used thionating reagent, caused the PDA to undergo a sequence of thionation, isomerization, and polymerization reactions [42]. To prove that the SCMP was polymerized, the solid-state ^13^C NMR spectra of the SCMP were obtained, as shown in Fig. S2. ^13^C peaks for the SCMP at 138.5, 129.7, and 123.7 ppm were detected, which are attributed to the C–S, C_C–S_–C–C_C–H_, and C–H bonds, respectively, indicating that the polymerization occurred as expected. Additionally, there was a peak near 175 ppm, which is attributed to the unreacted anhydride C (C_C=O_) from the PDA monomer. The FTIR spectra were also obtained, to confirm the polymerization. In Fig. S3, the FTIR absorption bands at 1771 and 1858 cm^−1^ are attributed to C=O vibration in the PDA monomer. After the polymerization, these absorption bands disappeared, and new absorption bands at 1612 and 1438 cm^−1^ emerged, which are assigned to the newly formed C=C bond [42]. To measure the carbon content, the TGA curve of the SCMP in N_2_ was obtained, as shown in Fig. S4. Considering the relatively low thermostability of the thiophene and sulfonate group compared with the carbon skeleton, the large weight loss during carbonization is ascribed to the decomposition of these functional groups. Finally, the SCs were obtained via the pyrolysis of the SCMP at different temperatures in N_2_. Samples carbonized at 600, 700, 800, and 900 °C are denoted as SC-600, SC-700, SC-800, and SC-900, respectively.

**Fig. 1:**
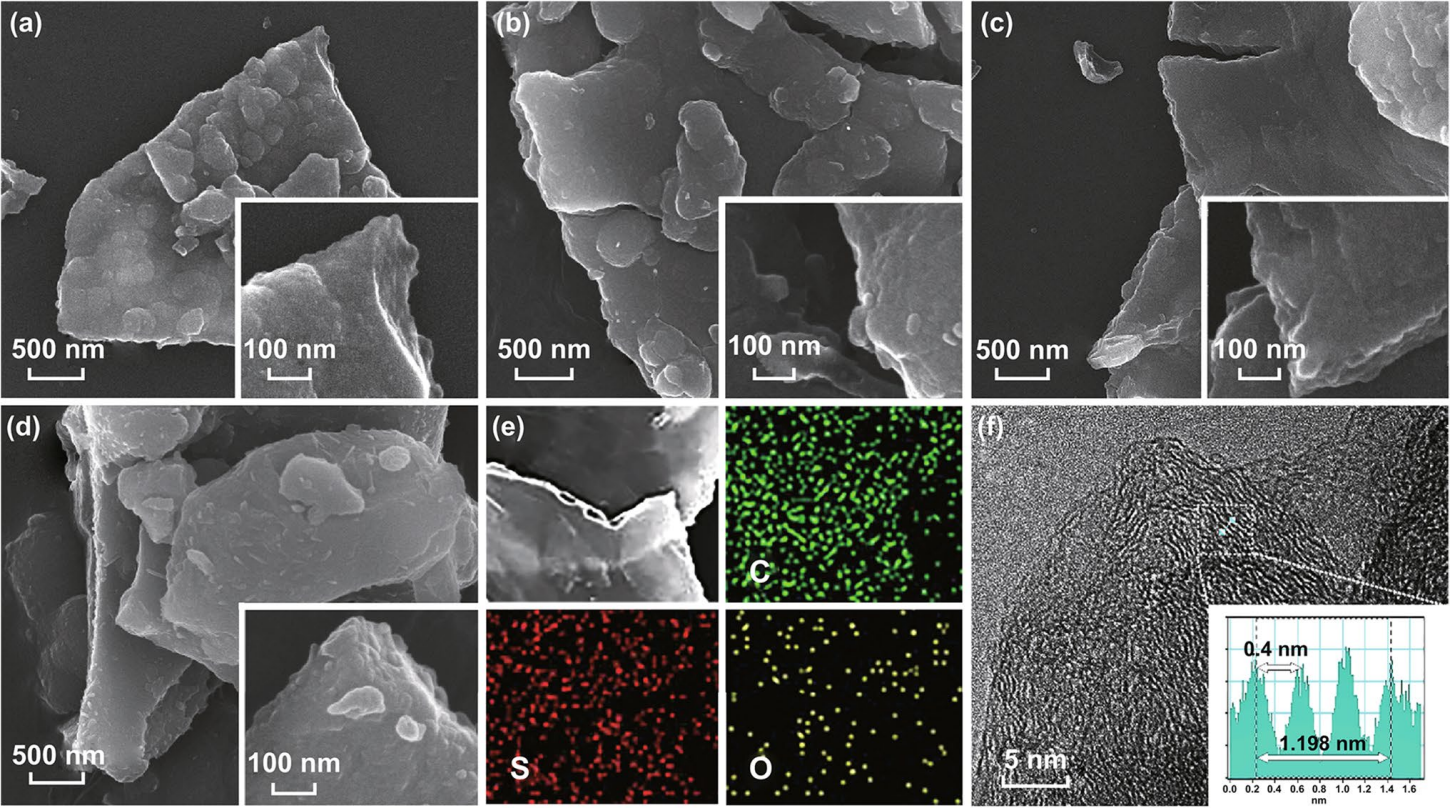
SEM images of **a** SC-600, **b** SC-700, **c** SC-800, and **d** SC-900. The insets show the corresponding magnified SEM images. **e** EDS elemental mapping of SC-800. **f** HRTEM image of SC-800. The inset shows the average interlayer distance at the electronic scale

Figure 1a–d shows SEM images of SC-600, SC-700, SC-800, and SC-900. All the SCs exhibited similar structures, indicating the stability of the SCMP-converted carbon matrix. To analyze the distribution of S in the SCs, energy-dispersive X-ray spectroscopy (EDS) elemental mapping was performed, as shown in Fig. 1e. We clearly observed that S was homogeneously distributed in SC-800, indicating that S was doped in the SCs. Figure 1f presents a high-resolution TEM (HRTEM) image of SC-800. As shown, a disorder interlayer distance was detected. After the disposition of the micro-region (inset in Fig. 1f), the average interlayer distance was 0.40 nm, which is larger than that for graphite. HRTEM images of SCs carbonized at other temperatures are displayed in Fig. S5, which all exhibited an expanded average interlayer distance of approximately 0.4 nm. The expanded interlayer distance of the SCs was expected to facilitate the insertion of Na ions into the layers [43].

**Fig. 2:**
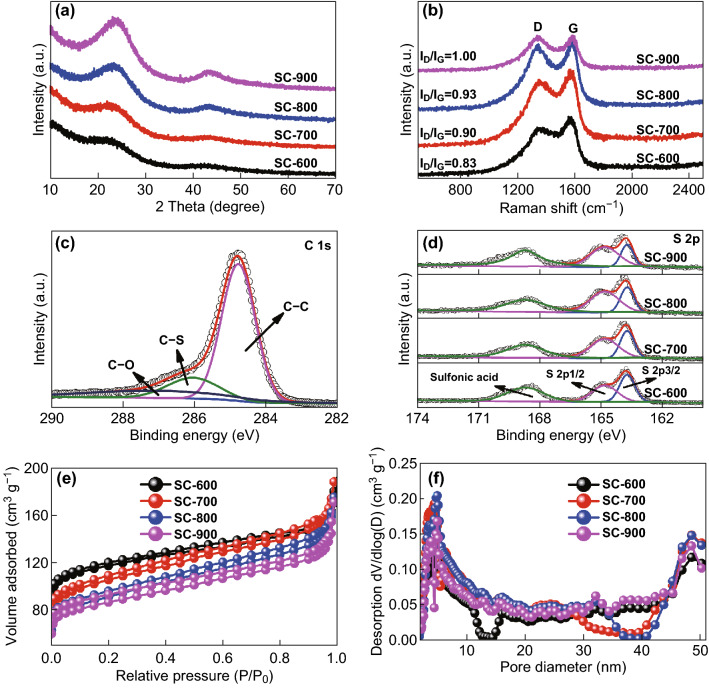
**a** XRD patterns and **b** Raman spectra of SC-600, SC-700, SC-800, and SC-900. **c** XPS C 1s profile of SC-800. **d** XPS S 2p profiles. **e** Nitrogen sorption isotherms and **f** pore-size distributions of SC-600, SC-700, SC-800, and SC-900

Owing to the larger covalent diameter of S compared with C, the substitution of C by S in the carbonaceous material increased the spacing between adjacent sheets, as indicated by HRTEM [9, 44]. To confirm this, the XRD patterns of the SCs were obtained, as shown in Fig. 2a. All the samples exhibited two broad peaks around 23.5° and 43.2°, corresponding to the (002) and (100) crystal faces of graphite, respectively [44, 45]. Using Bragg’s equation, the interlayer spacing (*d*_002_) of the samples was calculated as ~ 0.4 nm, which is consistent with the HRTEM results. This large interlayer spacing accelerated the diffusion of Na ions in the battery system, enhancing the electrochemical utilization. Figure 2b shows the Raman spectra of the SCs. Two bands centered at 1350 and 1590 cm^−1^ are observed, which are the D-band and G-band of C, respectively [46, 47]. Generally, the D-band represents the disorder carbon, and the G-band is related to the graphitic carbon. Hence, the intensity ratio of the D-band to the G-band (*I*_D_/*I*_G_) indicates the degree of disorder for carbon materials. *I*_D_/*I*_G_ was calculated as 0.83, 0.90, 0.93, and 1.00 for SC-600, SC-700, SC-800, and SC-900, respectively. These values indicate that more defects were generated at a higher temperature, providing more active sites for Na-ion accommodation [48].

To investigate the compositions of the SCs, XPS was performed. The S contents in SC-600, SC-700, SC-800, and SC-900 were 9.79, 8.69, 6.25, and 4.89 at%, respectively, indicating a declining trend with an increase in the carbonization temperature. Figure 2c shows the high-resolution C 1s spectrum of SC-800, which can be deconvoluted into three peaks. Among them, the spectrum is principally constituted by the C–C bond, indicating that the SCMP was carbonized after the pyrolysis. The other two peaks are attributed to the C–S and C–O bonds, indicating that the S atoms were linked tightly to the SCs. Figure 2d shows the high-resolution S 2p spectra of SC-600, SC-700, SC-800, and SC-900. After fitting, all the spectra could be deconvoluted into three peaks. Among them, the two peaks at 163.7 and 164.8 eV are attributed to S 2p_3/2_ and S 2p_1/2_, respectively, for the –C–S–C– covalent bond of thiophene S. The other peak at 168.4 eV corresponds to the C–SO_*x*_–C (*x* = 2–4) group, confirming that S was successfully incorporated into the carbon skeletons [9, 49, 50]. Notably, O was also present in the doped samples (as shown in Fig. S6), which should come from the unreacted anhydride C (as shown in Fig. S2). The doped O mainly bonded with S, forming a sulfonate group, as confirmed by the C–SO_*x*_–C (*x* = 2–4) group observed in Fig. 2d. This group may have increased the interlayer distance of the SCs owing to its relatively large size. The O contents in SC-600, SC-700, SC-800, and SC-900 were 14.2, 11.64, 9.19, and 8.20 at%, respectively, indicating a declining with the increasing carbonization temperature, similar to that of the S content.

Figure 2e, f shows the N_2_ sorption isotherms and pore-size distributions of the samples. According to the International Union of Pure and Applied Chemistry (IUPAC) classification, the isotherms of SCs (Fig. 2e) exhibit typical type IV curves with a hysteresis loop located in the relative pressure range of 0.1–0.9, indicating that all SCs have a mesoporous structure. Calculations using the BET model indicated that the specific surface areas of SC-600, SC-700, SC-800, and SC-900 were 446.1, 388.6, 331.4, and 320.5 m^2^ g^−1^, respectively. The BET specific surface area of the SCs decreased with the increasing pyrolysis temperature. This phenomenon was due to the reduction in the S doping content with the increasing pyrolysis temperature, which resulted in the decrease in structural defects in the SCs [51]. The pore-size distributions of the PCSs were analyzed via nonlocal density functional theory calculations, as shown in Fig. 2f. As expected, the pores of the SCs were mainly distributed in the mesoporous range, from 2 to 10 nm. Owing to the similar mesoporous structures of the SCs, a larger specific surface area of SCs provided a larger contact interface area between the electrode and the electrolyte [52, 53].

### Electrochemical Performance

**Fig. 3:**
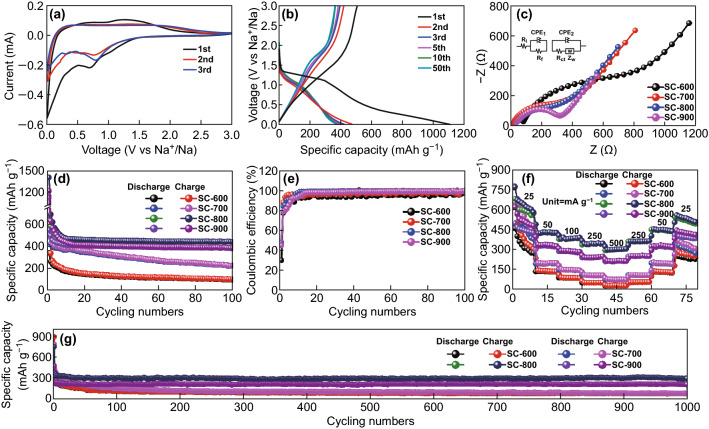
**a** CV curves and **b** charge/discharge profiles of SC-800. **c** Electrochemical impedance spectra, **d** cycling performance, **e** Coulombic efficiencies, **f** rate performance, and **g** long-term cycling performance of SC-600, SC-700, SC-800, and SC-900

To evaluate the electrochemical performance, the initial three CV curves of SC-800 were obtained, as shown in Fig. 3a. In the first cycle, a large irreversible cathodic peak was observed at approximately 0.8 V, which was due to the formation of a solid–electrolyte interphase (SEI) layer [9]. Additionally, a redox couple located at 1.1/1.8 V was observed, which is ascribed to the redox reaction of doped S in SC-800. This phenomenon is similar to those observed in previously reported Na-S batteries [54, 55]. Owing to the electrochemical activity of the covalently bonded S, our SCs could accommodate more Na ions, improving the reversible capacity [56]. Figure 3b displays the galvanostatic charge/discharge profiles of the SC-800 electrode at a current density of 50 mA g^−1^. Visible plateaus around 1.1/1.8 V are observed, corresponding to the redox reaction. After the initial three cycles, the specific capacity of SC-800 changed little.

Figure 3c shows the EIS spectra of the SCs electrodes, which were obtained after 100 cycles at 50 mA g^−1^. The Nyquist plots of the SCs electrodes were composed of an indistinct semicircle, a large semicircle, and a sloping line in the high-, medium-, and low-frequency regions, respectively. Among them, the indistinct small semicircle was related to the resistance of the SEI layer and the constant-phase element (CPE1). The large semicircle is attributed to the charge-transfer resistance (*R*_ct_) and CPE_2_. The sloping line corresponds to the Warburg impedance (*Z*_w_) stemming from the diffusion of Na ions. (The corresponding equivalent circuit of the EIS spectra is shown in Fig. 3d.) The *R*_ct_ values were 411.8, 255.9, 183.1, and 222.5 Ω for SC-600, SC-700, SC-800, and SC-900, respectively, which were obtained from the fitting results based on the equivalent circuit model shown in Fig. 3c. SC-800 had the lowest *R*_ct_, which facilitated the charge transfer and was thus beneficial to the Na-ion storage performance.

Figure 3d, e shows the cycling performance and corresponding Coulombic efficiencies of the SCs. All the SCs exhibited capacity fading in the initial several cycles, which is ascribed to the formation of the SEI layer and reactions between Na ions and surface functional groups or adsorbed molecules [57]. After the initial several cycles, the Coulombic efficiencies of all the electrodes improved to nearly 99%, indicating that the SCs electrodes had excellent reversibility for Na-ion storage. Among all the samples, the SC-800 electrode exhibited the highest stable reversible capacity of 440 mAh g^−1^ at 100 mA g^−1^ after 100 cycles, while the reversible capacities of SC-600, SC-700, and SC-900 electrodes were 97, 224, and 384 mAh g^−1^, respectively. Although the S content in the SCs decreased with an increase in the temperature (as indicated by XPS), leading to the reduction in the reversible capacity [48], the high *I*_D_/*I*_G_ ratio and low *R*_ct_ of SC-800 indicated that the disordered structure with rich active sites offered insertion/deinsertion of Na ions and enhanced the electron-transfer ability [58, 59]. Therefore, SC-800 exhibited a superior Na-ion storage capability.

**Fig. 4:**
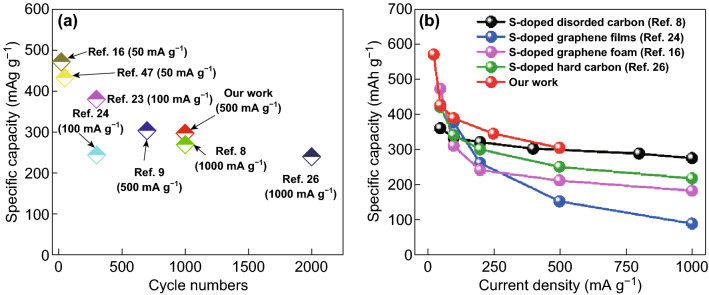
**a** Long-term cycling performance and **b** rate performance of other S-doped C-based materials for comparison

To further evaluate the electrochemical performance of the SCs, the rate performance was examined, as shown in Fig. 3f. The results indicated that the SC-800 electrode delivered reversible capacities of 570, 426, 389, 344, and 304 mAh g^−1^ at 25, 50, 100, 250, and 500 mA g^−1^, respectively. Thus, SC-800 exhibited superior rate performance to the other samples. When the current density recovered to 25 mA g^−1^, the capacity returned to 534 mAh g^−1^, indicating the excellent recoverability of the electrode. The excellent rate capability and recoverability of SC-800 were mainly attributed to the S doping, which increased the electrical conductivity and facilitated Na-ion transport by increasing the interlayer spacing [22]. The long-term cycling performance of the SCs was evaluated at a current density of 500 mA g^−1^, as shown in Fig. 4a. The reversible capacity of SC-800 reached 378 mAh g^−1^ in the first cycle and was stabilized at 297 mAh g^−1^ after 1000 cycles, which was significantly higher than those of SC-600 (76 mAh g^−1^), SC-700 (84 mAh g^−1^), and SC-900 (212 mAh g^−1^). To assess the Na-ion storage performance of our SCs, previously reported S-doped carbons were examined for comparison, as shown in Fig. 4a, b. Our SCs exhibited excellent rate performance, a high reversible capacity, and a stable cycling life, outperforming the other S-doped carbon materials.

**Fig. 5:**
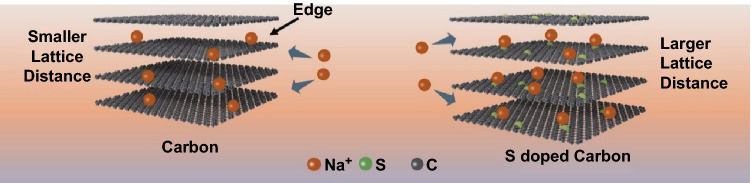
Possible Na-ion storage mechanism for SCs

The SCs exhibited better Na-ion storage performance than previously reported carbon materials without S doping [60, 61]. The reason for this was analyzed as follows. According to calculations performed by Yu et al. [61], the lattice distance of carbon is increased by S doping, which is consistent with our TEM results in Fig. 1f. Because of the small lattice distance, the adsorption energy between carbon and Na ions was 0.365 eV, illustrating that the adsorption of Na ions in the interior of carbon was unstable, and the capacity mainly came from the edge of carbon [61]. After the doping, the lattice distance was increased, and the adsorption energy between the S-doped carbon and Na ions was enhanced (–0.216 eV), improving the stability of Na-ion adsorption [61]. The corresponding schematic is shown in Fig. 5.

**Fig. 6:**
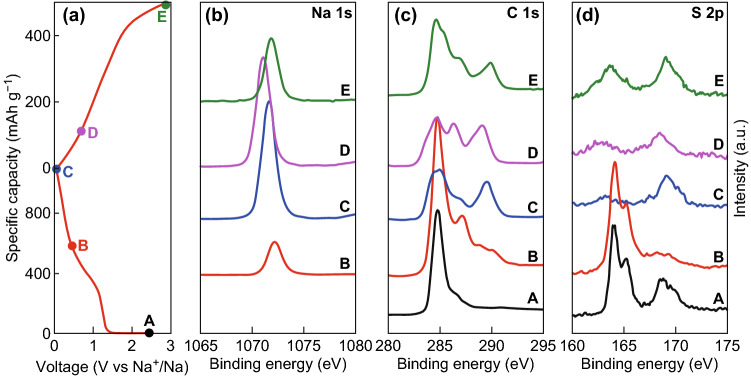
Ex situ XPS profiles of SC-800 in different states during the initial charge/discharge cycle: **a** corresponding voltage states; **b** Na 1s, **c** C 1s, and **d** S 2p XPS profiles

To further illustrate the reaction mechanism of the SCs for Na-ion storage, ex situ XPS profiles of SC-800 in different charge/discharge states for the initial cycle were obtained, as shown in Fig. 6. There was a peak in the Na 2p spectra after discharging (Fig. 6b), indicating that Na ions were inserted in SC-800. A significant shift was observed after discharging, which is attributed to the different states of Na-ion insertion [62]. After charging to 3 V, the peak returned to its original position, indicating the reversibility of our SCs for Na-ion storage [62]. The Na 2p peak still existed after cycling, owing to the formation of the SEI layer. Figure 6c shows the XPS C 1s profiles of SC-800 in different states. Clearly, the intensity of the peak corresponding to the C–C bond was reduced after discharging to 0.005 V and then increased after charging to 3 V, indicating that our SCs had good reversibility for Na-ion storage. Additionally, a peak at 289.6 eV existed after discharging. This peak corresponds to the O–C=O bond and is attributed to the formation of organic matter from the SEI layer [63, 64]. To further investigate the capacity contribution from S, the XPS S 2p profiles were obtained, as shown in Fig. 6d. Thiophene S was transformed into oxidized S after discharging, indicating that the Na ions reacted with the doped S in the SCs, including the reversible part (Na_2_S) and irreversible part (Na_2_SO_4_). For the reversible part, Na ions reacted with C-S bonds, forming C-S-Na bonds. For the irreversible part, the reaction was mainly caused by the formation of the SEI layer. To further analyze the SEI layer, the Cl 2p XPS profile after the initial cycle was obtained, as shown in Fig. S7. Two peaks at 199.1 and 200.8 eV were observed, which were due to the Na–Cl bond, indicating the presence of NaCl in the SEI layer [65].

**Fig. 7:**
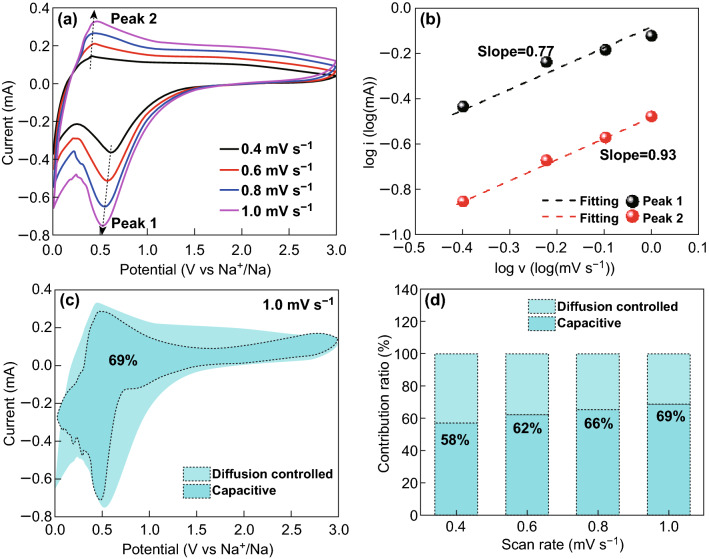
**a** CV curves of SC-800 at different sweep rates after cycling. **b** Relationship between log(*i*) and log(*v*) for SC-800. **c** Voltammetric response (1.0 mV s^−1^) for SC-800 and **d** contributions of the capacitive and diffusion-controlled charges at different sweep rates

To analyze the possible storage mechanism of the SCs electrodes for NIBs, the electrochemical kinetics for Na-ion storage were investigated. Figure 7a shows the CV profiles of SC-800 at different sweep rates ranging from 0.2 to 2 mV s^−1^. With increasing sweep rate, all the CV curves exhibited similar shapes, and slight shifts of the cathodic and anodic peaks were observed owing to the contribution of the capacitive characteristic. To analyze this characteristic, the relationship between the current and sweep rate was calculated according to the literatures (Eqs. 1 and 2) [66, 67]:

1 $$i = av^{b}$$

2 $$\log i = b\log v + \log a$$

where *i* and *v* represent the current density and sweep rate, respectively, and *a* and *b* are constants. According to the literature, the diffusion-controlled process is dominant if *b* is close to 0.5. Otherwise, the predominant mechanism is the surface-limited capacitive characteristic (*b* close to 1). Using Eq. 2, we calculated the *b* values from the slope of the graph of log *i* versus log *v*. The *b* values of peaks 1 and 2 were 0.77 and 0.93, respectively, as shown in Fig. 7b. These results indicate that the capacity of the SC-800 electrode was dominated by the capacitive contribution. To confirm the total contribution for the SC-800 electrode, the fractions of capacitor-like (*k*_1_*v*) and diffusion-controlled (*k*_2_*v*^1/2^) currents were distinguished at a fixed potential (*V*) according to Eq. 3 [68–70]:

3 $$i(V) = \, k_{1} v \, + \, k_{2} v^{1/2} ,$$

where *k*_1_ and *k*_2_ are constants. Equation 3 can be rewritten as Eq. 4:

4 $$i(V)/v^{1/2} = k_{1}v^{1/2} + k_{2}.$$

The values of *k*_1_ and *k*_2_ are easily calculated via plotting the fitting lines of *i*(*V*)/*v*^1/2^ and *v*^1/2^, and then the capacitive current *i*_*c*_(*V*) = *k*_1_*v* can be distinguished from the total measured current. For example, the capacitive current was compared with the tested current from the CV curves, as shown in Fig. 7c. The results indicated that the capacitive contribution to the capacity was 69% at a sweep rate of 1.0 mV s^−1^. Figure 7d shows the capacitive contribution of SC-800 at sweep rates of 0.4, 0.6, 0.8, and 1.0 mV s^−1^. The corresponding capacitive contribution ratios were 58%, 62%, 66%, and 69%. The capacitive contribution exhibited a rising trend with the increasing scan rate. The high capacitive contribution is ascribed to the short ion-diffusion length and rapid electron transfer and was responsible for the high-rate capability [70]. Because of the capacitive behavior, a large number of Na ions could be easily stored on the surface or near-surface sites of the electrode, resulting in excellent cycling stability [68].

## Conclusions

We successfully synthesized SCs from CMPs via a convenient, economical, and scalable method. Owing to the structural features provided by the CMP precursor, such as the large interlayer spacing, enhanced charge-transfer ability, and hierarchical pore distribution, SC-800 exhibited a high reversible capacity (440 mAh g^−1^ at 50 mA g^−1^), excellent rate performance, and superior cycling performance (297 mAh g^−1^ at a current density of 500 mA g^−1^ after 1000 cycles) for NIBs. The excellent performance of the SCs is attributed to the expanded lattice distance after S doping. Furthermore, we employed ex situ XPS to investigate the electrochemical reaction mechanism of the SCs during sodiation–desodiation, which can highlight the role of doped S for Na-ion storage.

## Electronic supplementary material

Below is the link to the electronic supplementary material.

Supplementary material 1 (PDF 711 kb)
