# Supplementary material for: High-Performance Na-Ion Storage of S-Doped Porous Carbon Derived from Conjugated Microporous Polymers
Source: Nanomicro Lett. 2019 Jul 17;11:60. doi: 10.1007/s40820-019-0291-z (PMC7770694; doi:10.1007/s40820-019-0291-z)
Supplement: Supplementary file 1 — Supplementary material 1 (PDF 711 kb) [file 40820_2019_291_MOESM1_ESM.pdf]

Supporting Information for

High-performance Na-ion Storage of S-doped Porous Carbon Derived from Conjugated Microporous Polymers

Yuquan Li[1, †], Bin Ni[1, †], Xiaodan Li[2], Xianghui Wang[1, *], Dafeng Zhang[3], Qingfei Zhao[5], Jinliang Li[2, *], Ting Lu[1, 4, *], Wenjie Mai[2], Likun Pan[1]

[1]Shanghai Key Laboratory of Magnetic Resonance, School of Physics and Materials Science, East China Normal University, 3663 N. Zhongshan Rd., Shanghai 200062, People's Republic of China

[2]Siyuan Laboratory, Guangdong Provincial Engineering Technology Research Center of Vacuum Coating Technologies and New Energy Materials, Department of Physics, Jinan University, Guangzhou, Guangdong 510632, People's Republic of China

[3]School of Materials Science and Engineering, Liaocheng University, Liaocheng, Shandong 252000, People's Republic of China

[4]Department of Chemical Engineering, School of Environmental and Chemical Engineering, Shanghai University, 99 Shangda Road, Shanghai 200444, People's Republic of China

[5]Testing and Analysis Centre, College of Chemistry and Materials Science, Shanghai Normal University, 100 Guilin Road, Shanghai 200234, People's Republic of China

†Yuquan Li and Bin Ni contributed equally to this work.

*Corresponding authors. E-mail: xhwang@phy.ecnu.edu.cn (Xianghui Wang); lijinliang@email.jnu.edu.cn (Jinliang Li); tlu@phy.ecnu.edu.cn (Ting Lu)

Supplementary Figures

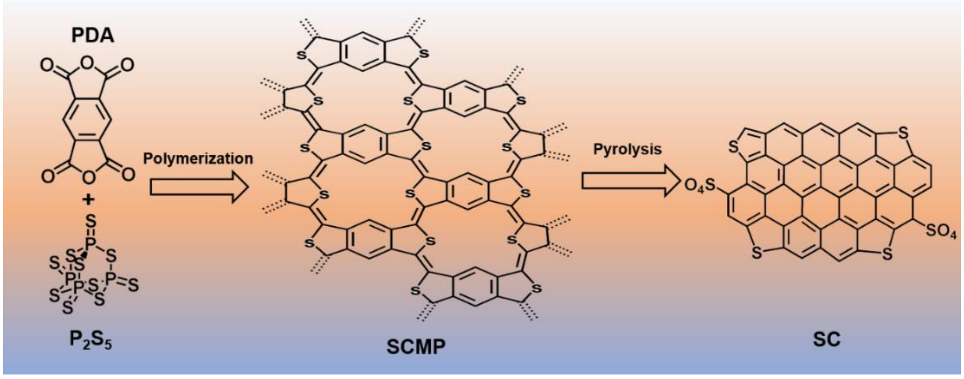

Fig. S1 Schematic of the synthesis process of SCs

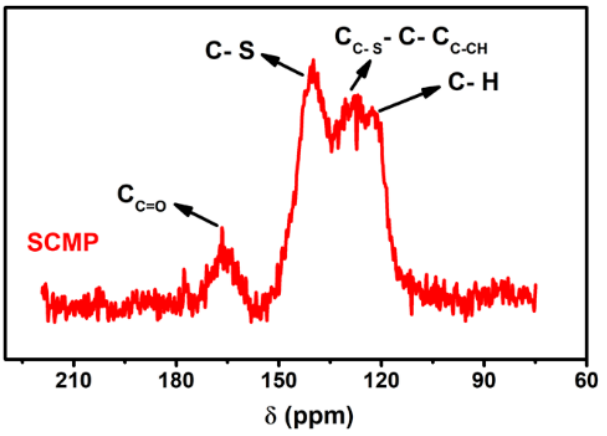

Fig. S2 Solid-state ^{13}C NMR spectra of SCMP

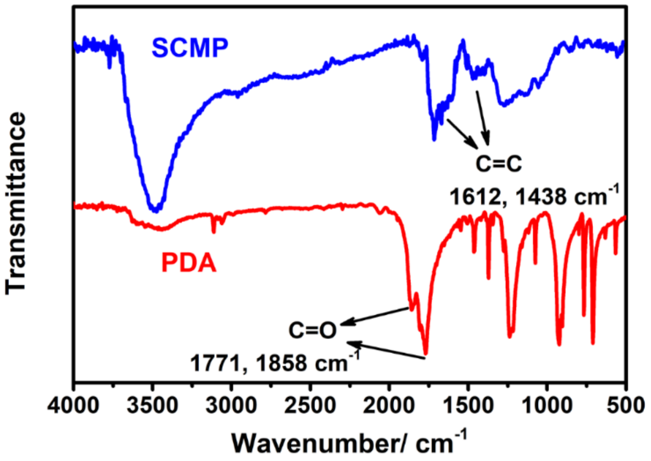

Fig. S3 FTIR spectra of PDA and SCMP

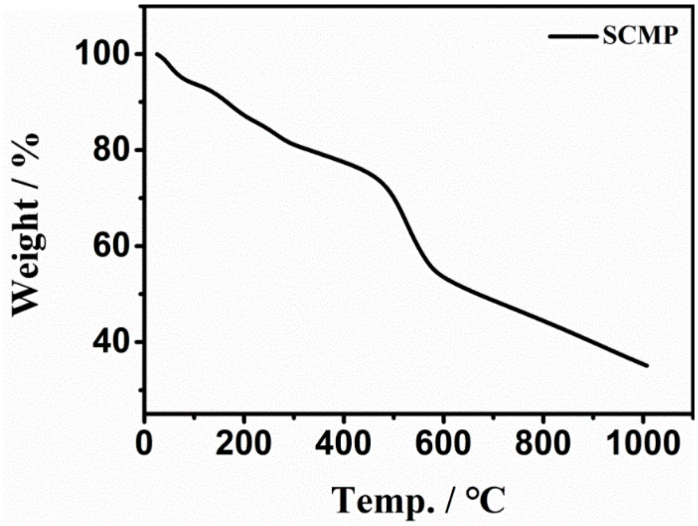

Fig. S4 TGA curve of SCMP in nitrogen atmosphere

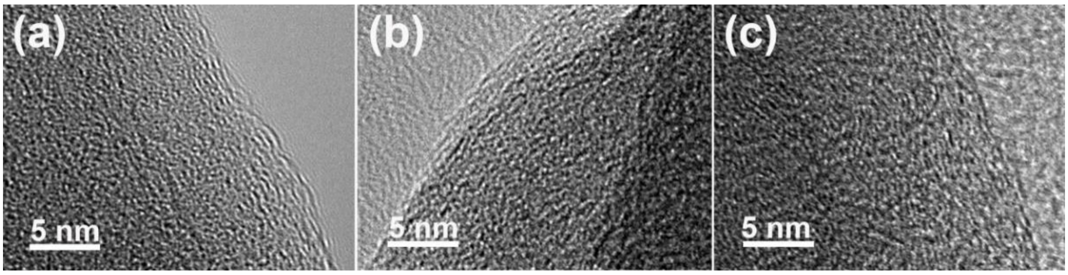

Fig. S5 HRTEM images of **a** SC-600, **b** SC-700, **c** SC-800, **d** SC-900

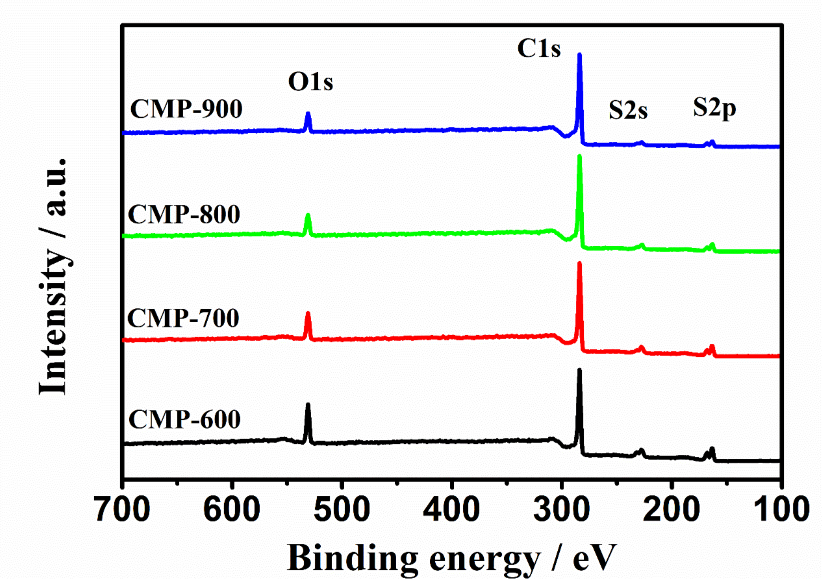

Fig. S6 XPS spectra of SCs

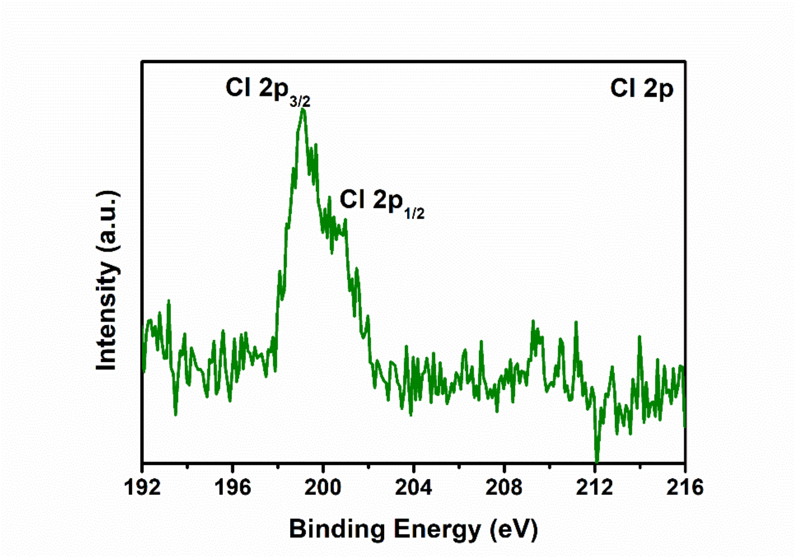

Fig. S7 Ex-situ Cl 2p XPS spectrum of SC-600 after initial cycle
